# A core UPS molecular complement implicates unique endocytic compartments at the parasite–host interface in *Giardia lamblia*

**DOI:** 10.1080/21505594.2023.2174288

**Published:** 2023-02-13

**Authors:** Erina A. Balmer, Corina D. Wirdnam, Carmen Faso

**Affiliations:** a Institute of Cell Biology, University of Bern, Bern, Switzerland; b Graduate School for Cellular and Biomedical Sciences, University of Bern, Bern, Switzerland; c Multidisciplinary Center for Infectious Diseases, University of Bern, Bern, Switzerland

**Keywords:** *Giardia lamblia*, virulence, unconventional protein secretion, interactome, peripheral endocytic compartments

## Abstract

Unconventional protein secretion (UPS) plays important roles in cell physiology. In contrast to canonical secretory routes, UPS does not generally require secretory signal sequences and often bypasses secretory compartments such as the ER and the Golgi apparatus. Giardia lamblia is a protist parasite with reduced subcellular complexity which releases several proteins, some of them virulence factors, without canonical secretory signals. This implicates UPS at the parasite–host interface. No dedicated machinery nor mechanism(s) for UPS in Giardia are currently known, although speculations on the involvement of endocytic organelles called PV/PECs, have been put forth. To begin to address the question of whether PV/PECs are implicated in virulence-associated UPS and to define the composition of molecular machinery involved in protein release, we employed affinity purification and mass spectrometry, coupled to microscopy-based subcellular localization and signal correlation quantification to investigate the interactomes of 11 reported unconventionally secreted proteins, all predicted to be cytosolic. A subset of these are associated with PV/PECs. Extended and validated interactomes point to a core PV/PECs-associated UPS machinery, which includes uncharacterized and Giardia-specific coiled-coil proteins and NEK kinases. Finally, a subset of the alpha-giardin protein family was enriched in all PV/PECs-associated protein interactomes, highlighting a previously unappreciated role for these proteins at PV/PECs and in UPS. Taken together, our results provide the first characterization of a virulence-associated UPS protein complex in *Giardia lamblia* at PV/PECs, suggesting a novel link between these primarily endocytic and feeding organelles and UPS at the parasite–host interface.

## Introduction

Unconventional protein secretion (UPS) is an umbrella term which defines secretion routes alternative to classic ER to Golgi secretion, guided by signal peptides [1,2]. To date, four UPS pathways have been formally described: protein secretion via self-sustained channel formation (UPS I), ABC transporter-mediated membrane passage (UPS II), vesicular export involving autophagy components (UPS III), and Golgi bypass of membrane proteins (UPS IV) [1,3,4]. UPS has also been reported in protist parasites [5–11]. There is evidence for UPS type II involving ABC transporters in both *Leishmania* [5–7] and *Plasmodium* [8]; evidence for UPS type III involving extracellular vesicles/exosomes is reported for *Trichomonas vaginalis* and *Tritrichomonas foetus* (recently reviewed in [9]). Exosome production in Leishmania was reported by Silverman and colleagues [10] while *Plasmodium* has been shown to produce extracellular vesicles at different life cycle stages [11]. However, the exact mechanisms for release of virulence and other parasite-derived factors, in most cases, have not been defined.

*Giardia lamblia* (*syn. Giardia intestinalis, Giardia duodenalis*) is a small-intestine protist parasite with a worldwide distribution. *Giardia* presents a simplified endomembrane system, with only five membrane-bound compartments, including an extensive ER, and no detectable Golgi apparatus [12–14] despite constitutive trafficking of secretory variant surface proteins [15–17]. The sole port of entry for fluid-phase material in the Giardia cell are the recently renamed [18] peripheral vacuoles/peripheral endocytic compartments (PVs/PECs), small (ca. 200 nm) highly polymorphic organelles [19–22,51]. These organelles present a clearly endocytic molecular complement composed of clathrin assemblies, endocytic adaptor and lipid-binding proteins, ESCRT components, and putative receptors [19–21]. PVs/PECs perform cycles of uptake and release of extracellular medium as they temporarily fuse with the plasma membrane [20,22].

Virulence at the Giardia-host interface is poorly understood, with secreted cysteine proteases (CPs) and their documented negative impact on host cells (disruption of cell–cell adhesion, microbiota and the mucus layer, apoptosis of epithelial cells alongside degradation of antibodies and α-defensins) as perhaps the best characterized example of virulence factors [23–27]. Recent reports document a large number of Giardia-derived proteins released in the extracellular space or found on the surface of Giardia cells [28–30], in both axenic and co-cultivation conditions, with mostly unclear roles in virulence. However, the striking feature of these data is that, in contrast to CPs which present unequivocal sequences for secretion, the vast majority of proteins detected in secretome and surface proteome analyses of Giardia trophozoites are predicted to be soluble, non-secretory and intracellular, thus implicating UPS in their release. Among them are notable examples such as enolase (ENO) and arginine deiminase (ADI). ENO is perhaps one of the most studied candidate UPS substrate for its role as putative virulence factor in several parasitic species, including *Plasmodium*, *Trichomonas,* and *Entamoeba* [31–34], although its role in pathogenesis is not fully understood [35,36]. In co-cultivation studies with intestinal epithelial cells and subsequent comparison to secretion in axenic cultures, Giardia-derived ENO and ADI were shown to be increased in the extracellular medium after incubation with host cells [28,36,37], with ENO also investigated as a candidate vaccination antigen for giardiasis [38]. In contrast to ENO, a more robust role for Giardia released ADI-mediated host arginine depletion has been defined, with a measurable negative impact on nitric oxide production, intestinal T-cell proliferation, and dendritic cell cytokine secretion [39,40]. Finally, alpha 1-giardin, a reportedly cytoskeletal component with strong immunogenic properties, is under consideration as an excellent candidate for vaccination [41,42].

Despite the fact that the Giardia-host interface is populated by many soluble non-secretory proteins, some of them with documented virulence function, no dedicated machinery nor mechanism(s) for UPS in Giardia are currently known. Speculations on the involvement of PV/PECs have been put forth previously [58–60]. We therefore hypothesized that, with PV/PECs at the host–pathogen interface and in direct communication with the extracellular environment, a dedicated UPS machinery could be found at PV/PECs for the release of unconventionally secreted putative and confirmed virulence factors.

To test this hypothesis, we selected 11 putative UPS substrates with no detectable secretory signals and transmembrane domains, and characterized them in terms of subcellular localization and protein interaction partners. We employed immuno-fluorescence assays, including co-labeling experiments and quantification of signal overlap, on Giardia trophozoites expressing epitope-tagged putative UPS substrate. This was followed by co-immunoprecipitation (co-IP) and mass spectrometry-based protein identification, to define and expand the UPS interactome. In line with the presented hypothesis, a subset of the selected putative virulence factors were found to localize to PVs/PECs in a tight interactome network with specific PV-PECs-associated NEK kinases and coiled-coil proteins. This network includes several alpha giardins, annexin homologs [43,44] which also localize to PV/PECs. These data shed light on a novel link between unique endocytic compartments and virulence-associated UPS protein complexes at the parasite–host interface in *Giardia lamblia*.

## Results

### Selected putative UPS substrates localize to PV/PECs

As a first step toward testing the hypothesis that UPS in Giardia is linked to PV/PECs, we searched through reported datasets derived from secretome and surface proteome analyses [28–30]. We selected 11 putative UPS substrates (Table 1) based on criteria extracted from GiardiaDB and other sources:

- Detection in the extracellular space (according to [28–30])
- Absence of predicted signal peptide using prediction algorithms (SignalP using SP-HMM/SP-NN [45]
- Absence of predicted transmembrane domains (detected by TMHMM [46]
- Availability of transcriptomics data [47–50].

**Table 1.** Selected putative UPS substrates including histone H2A as a negative control for secretion and peroxiredoxin-1 as a predicted canonically secreted protein. Open reading frame (ORF) numbers and protein names are followed by the secretome or surface proteome study they were identified in [28–30]. The third column indicates if the protein is predicted to carry a signal peptide for secretion (SP) and/or a transmembrane domain (TMD), as retrieved from GiardiaDB. The last column extracts transcriptomics data from two RNA seq studies on *G. lamblia* WB-A [47,48] with transcript abundance expressed in TPM (transcripts per million).

ORF and protein name	Found in secretome (sec.) or surface proteome (sur.)	SP or TMD	Unique transcript expression RNA seq WBA (Franzén et al.2013/Tolba et al 2013) [TPM]
GL50803_11118 Enolase	sec. (Dubourg et al. 2017, Ma’ayeh et al. 2017)	No	623.14/1019.83
GL50803_112103 Arginine deiminase	sec. (Ma’ayeh et al. 2017)	No	1100.54/1715.93
GL50803_4812 beta giardin	sur. (Davids et al. 2019) and sec. (Dubourg et al. 2017, Ma’ayeh et al. 2017)	No	2473.07/0
GL50803_11654 alpha 1-giardin	sur. (Davids et al. 2019) and sec. (Dubourg et al. 2017, Ma’ayeh et al. 2017)	No	783.76/2887.52
GL50803_17153 alpha 11- giardin	sur. (Davids et al. 2019) and sec. (Dubourg et al. 2017, Ma’ayeh et al. 2017)	No	398.57/1099.9
GL50803_16453 carbamate kinase	sur. (Davids et al. 2019) and sec. (Ma’ayeh et al. 2017)	No	1535.07/729.65
GL50803_17327 Xaa-Pro dipeptidase	sec. (Ma’ayeh et al. 2017)	No	212.2/39.26
GL50803_3910 hypothetical	sec. (Ma’ayeh et al. 2017)	No	157.87/236.14
GL50803_112304 EF1-alpha	sec. (Dubourg et al. 2017, Ma’ayeh et al. 2017)	No	161.61/76
GL50803_12091 Macrophage migration inhibitory factor	sec. (Dubourg et al. 2017)	No	140.06/16.53
GL50803_6687 GAPDH	sec. (Ma’ayeh et al. 2017)	No	258.27/982.49
GL50803_27521 Histone H2A (nuclear reference dataset)	No	No	340.53/461.96
GL50803_15383 Peroxiredoxin-1-paralog (canonical secretion reference dataset)	sur. (Davids et al. 2019) and sec. (Ma’ayeh et al. 2017)	SP	175.09/183.86

**Figure 1.**
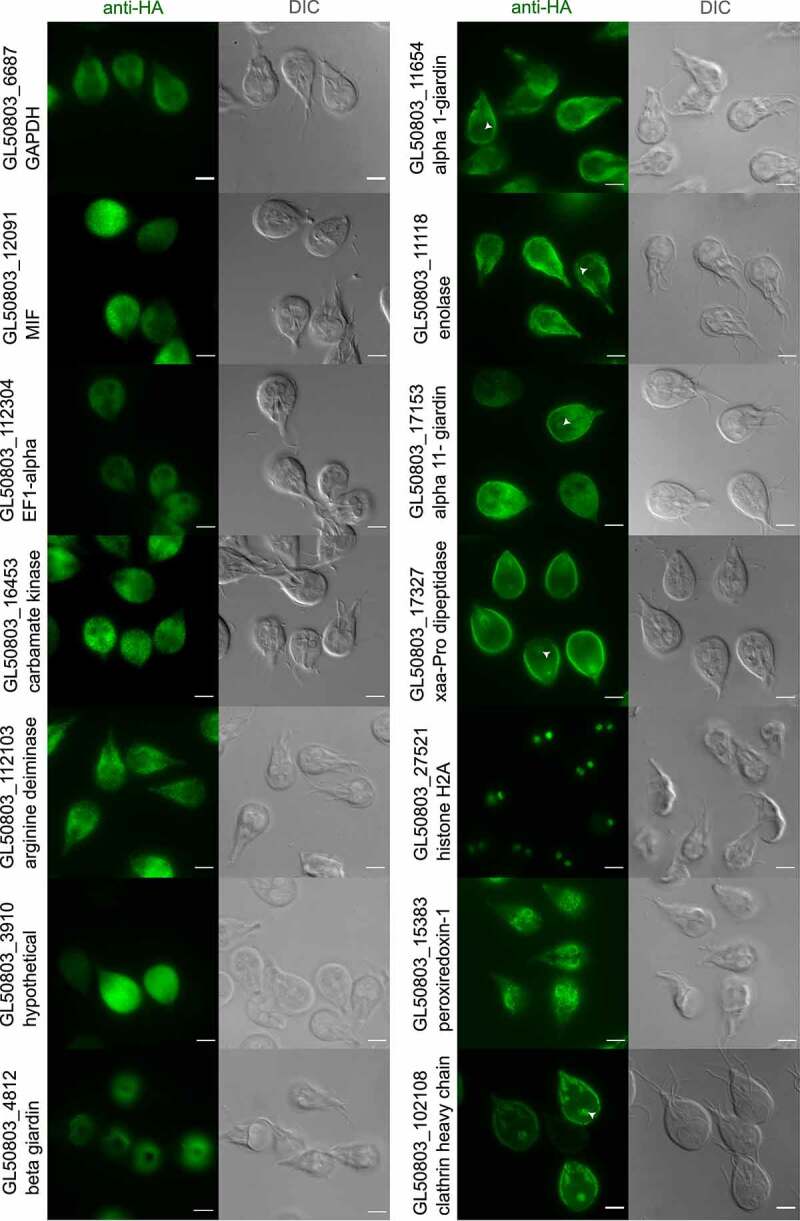
Selected putative UPS substrates localize to PV/PECs. Representative widefield light microscopy images of antibody-labeled HA epitope tagged UPS substrates and controls, expressed in Giardia trophozoites (anti-HA panels) including DIC images (Differential Interference Contrast). The first six transgenic lines on the left (GL50803_112103 Arginine deiminase, GL50803_12091 Macrophage migration inhibitory factor (MIF), GL50803_3910 hypothetical protein, GL50803_16453 carbamate kinase, GL50803_112304 TEF1 alpha, GL50803_6687 GAPDH (Glyceraldehyde 3-phosphate dehydrogenase)) show a cytosolic distribution, while GL50803_4812 beta-giardin shows localization to the ventral disc. The first four transgenic lines on the right (GL50803_17327 Xaa-Pro dipeptidase, GL50803_11654 alpha 1-giardin, GL50803_17153 alpha 11-giardin, GL50803_11118 Enolase) show a proximity to PV/PECs. GL50803_27521 Histone H2A localizes to nuclei while GL50803_15383 Peroxiredoxin-1 shows an ER localization pattern. GL50803_102108 *Gl*chc is included as a *bona fide* PV/PECs localized protein [18–20]. The PV/PECs-enriched bare zone is highlighted with a white arrowhead. Scale bars: 5 µm.

Stably transfected *G. lamblia* WB-A transgenic lines expressing epitope-tagged variants of all selected putative UPS substrates were generated, including transgenic lines expressing either epitope-tagged histone H2A or peroxiredoxin-1-paralog variants as references for non-UPS proteins, nuclear and secretory, respectively. Analysis by immunofluorescence assays (IFA) followed by widefield microscopy of all transgenic lines show that, in contrast to epitope-tagged histone2A and peroxiredoxin-1-paralog which localize to the nuclei and to the ER, respectively, and to beta-giardin which localizes to the ventral disc, all other selected UPS protein substrates localize to the cytosol, as predicted (Figure 1). However, Xaa-Pro dipeptidase, alpha 1-giardin, alpha 11-giardin and enolase show additional deposition in close proximity to PV/PECs, as indicated by signal at the periphery of the cell and in the “bare zone” between nuclei [51–53] similar to *Gl*CHC, a previously characterized *Giardia* protein known to accumulate at PV/PECs [19,20,54]. These proteins and their respective transgenic lines were selected for further investigation to better define subcellular localization of the corresponding reporter variants.

### Confocal microscopy and signal overlap analysis confirms UPS substrates at PV/PECs

**Figure 2.**
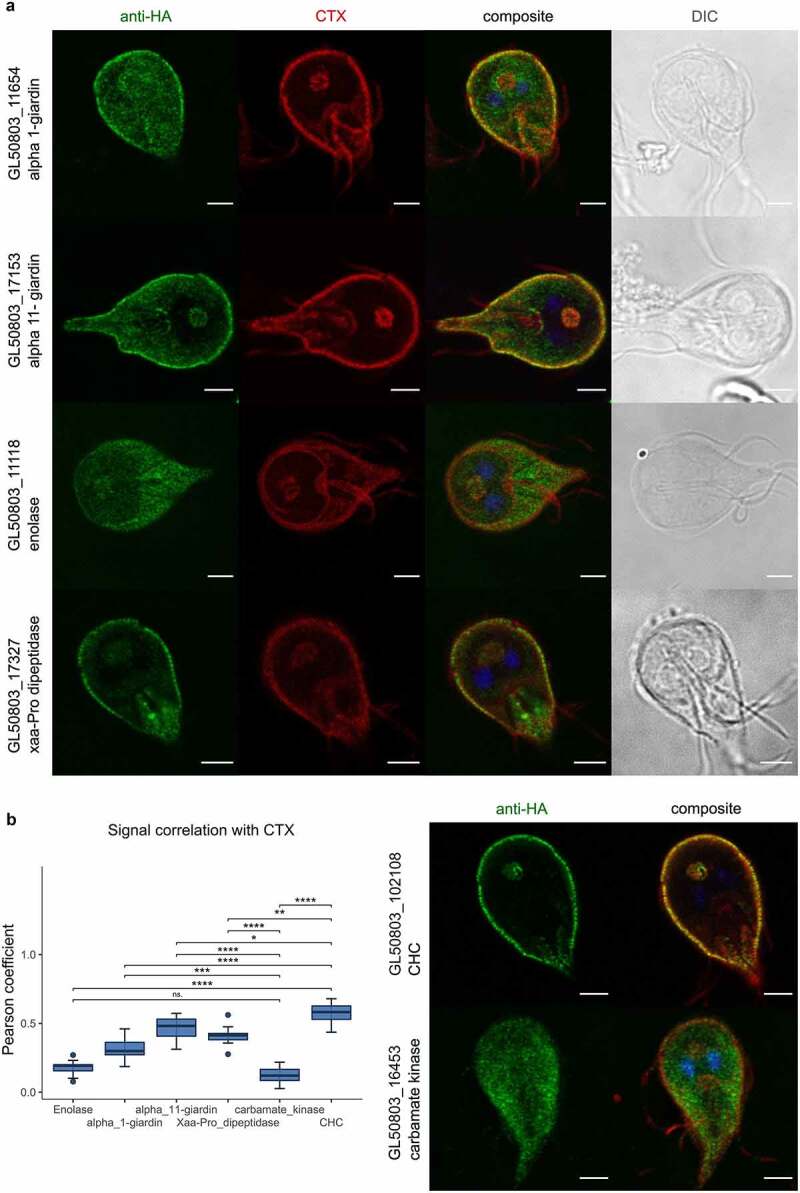
Confocal microscopy and signal overlap analysis confirms UPS substrates at PV/PECs. **(a)** Confocal microscopy images of antibody-labeled HA epitope tagged proteins expressed in transgenic Giardia trophozoites (anti-HA panels in green) including co-labelling with the PV/PECs membrane marker cholera toxin B, Alexa Fluor™ 594 Conjugate in red (CTX), a composite image of the two panels and the nuclei stained with DAPI and a DIC image. **(b)** Signal correlation for each transgenic line was quantified calculating the Pearson coefficient (*N* = 10). A *t*-test was performed to compare all values to the cytosolic control carbamate kinase and GlCHC as a PV/PECs marker. Significance levels are indicated by asterisks. The data are displayed as a boxplot and n.S. indicates a non-significant difference (*p*-value >0.05). The right panel shows representative single cell images of the control constructs. Scale bars: 3 µm.

Since widefield microscopy analysis of transgenic antibody-labeled Giardia lines expressing tagged variants of Giardia proteins Xaa-Pro dipeptidase, alpha 1-giardin, alpha 11-giardin, and enolase revealed localization in proximity to PV/PECs, we applied confocal microscopy analysis following immunofluorescence assays (IFA; Figure 2(a)). Giardia cells expressing Xaa-Pro dipeptidase, alpha 1-giardin, alpha 11-giardin, and enolase present signal concentrated at the cell periphery and in the bare-zone between the nuclei.

As a measure of the degree of association of reporter Xaa-Pro dipeptidase, alpha 1-giardin, alpha 11-giardin, and enolase variants to PV/PECs, co-labeling experiments for the epitope-tagged variants with the plasma/PV membrane marker cholera toxin-b (CTX [20,55] (Figure 2(a)) and subsequent signal correlation analysis using a dedicated macro script was performed (Figure 2(b) and Supplementary data 2). Pearson coefficient values were calculated for each line (*N* = 10) (Supplementary table S2), including control cell lines expressing epitope-tagged variants of *Gl*CHC and Giardia carbamate kinase (*Gl*CK), for a predominantly PV/PECs or cytosolic deposition pattern, respectively (Figure 2(b), right panel). A moderate positive correlation is found for the tested lines, accounting for the substantial cytosolic protein pool for each reporter. Signal overlap analysis (Figure 2(b), box plot) shows that enolase deposition presents the lowest correlation to CTX signal, with no significant deviation from the predominantly cytosolic *Gl*CK control line, despite clear PV/PECs association. However, signal overlap analysis for cells expressing Xaa-Pro dipeptidase, alpha 1-giardin, and alpha 11-giardin variants indicates how these proteins differ significantly from *Gl*CK and approach *Gl*CHC as our reference marker for PV/PECs association.

### UPS substrates at PVs/PECs participate in a core molecular complement

Alongside subcellular localization studies, we proceeded to define a UPS interactome for all selected putative UPS substrates.

To do this, epitope-tagged variants for each protein were expressed, extracted from transgenic *Giardia* cells, and used as affinity handles for co-immunoprecipitation (co-IP) in both limited cross-linking (to stabilize transient protein complexes) and native conditions, followed by mass spectrometry-based protein identification. As a qualitative control for background antibody binding, we used non-transgenic wild-type cell extracts in both co-IP conditions. Furthermore, as reference for known non-UPS associated proteins, we included the HA-tagged histone 2A (nuclear) and peroxiredoxin-1 paralog (ER) reporter lines. Proteins detected in these control/reference datasets were not subtracted prior to more in-depth analyses so as not to impact the enrichment values for putative *bona fide* interactors (Supplementary tables 6–9). Furthermore, these proteins are highlighted as possible contaminants since they were also found in wild-type datasets. The 10 most enriched interactors identified in each co-IP dataset, as measured by average relative iBAQ (intensity-based absolute quantification) values across two independent biological replicates are listed in Supplementary table 3 (Table 1: with cross-linking; Table 2: without cross-linking; proteins found in wild-type datasets are highlighted in gray).

**Table 2.** Top 10 most abundant interactors of selected NEK kinases and coiled-coil proteins as measured by co-IP in limited cross-linking conditions.

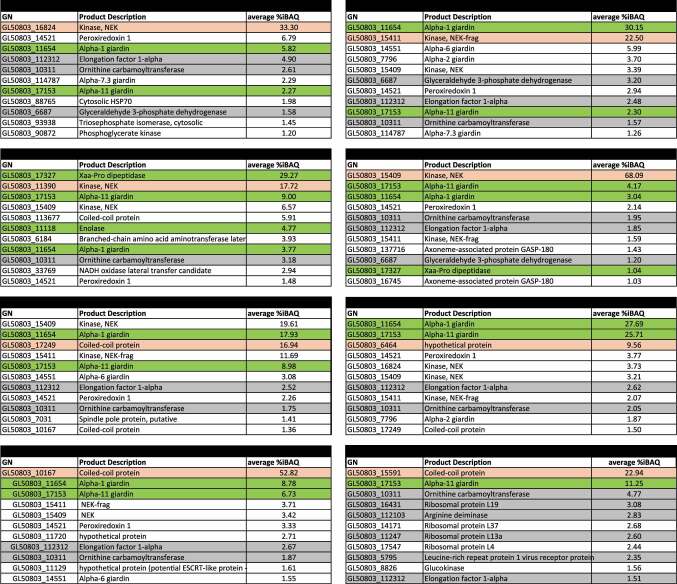

The top 10 interactors of the investigated coiled-coil proteins and NEK kinases including the GL50803_6464 hypothetical protein which shows structural similarities to a coiled-coil protein, are listed. Interactors are sorted according to average relative iBAQ values. Highlighted in orange is the affinity handle protein for each co-IP dataset. Highlighted in green are the PV/PECs-associated putative UPS substrates serving as “baits” in the first round of co-IP which pulled down the corresponding NEK kinase/coiled-coiled protein. Highlighted in gray are all proteins found in wild-type cross-linked co-IP experiments (Supplementary table 8). GN: gene number.

Histone H2A is mainly associated with other histones (Supplementary table 3, pale blue) while the interactome of Peroxiredoxin-1 mainly consists of Protein disulphide isomerases (PDI) and other *bona fide* ER resident proteins (Supplementary table 3, pale green), as expected for a predicted canonically secreted protein carrying a secretory signal sequence. In stark contrast, NEK kinases and coiled-coil proteins are predominant in PV/PECs-associated data sets and absent from the list of 10 most enriched interactors for both UPS cytosolic substrates and controls, in both types of co-IP setting (Supplementary table 3, pale yellow).

Native co-IP confirmed 28 of the 32 displayed interaction links found by co-IP in limited crosslinking conditions. Notably, native co-IP of carbamate kinase employed as a cytosolic non-PV associated UPS substrate yields a dataset which mirrors native co-IP of a non-transgenic sample (Supplementary table 9). This is in stark contrast to the datasets generated from co-IP of enolase, alpha 1 and alpha 11-giardin and Xaa-Pro dipeptidase, which present a clear enrichment for coiled-coil proteins and NEK kinases (Supplementary table 9).

**Figure 3.**
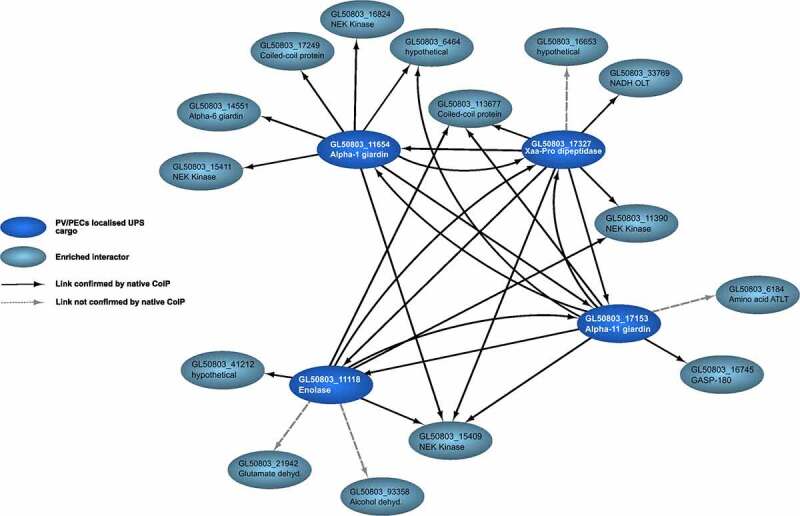
UPS substrates at PV/PECs participate in a core molecular complement. Depicted are the ten most enriched interactors in co-IP experiments for each PV-associated UPS substrate, not found in wild-type control samples (in dark blue, GL50803_17327 Xaa-Pro dipeptidase, GL50803_11654 alpha 1-giardin, GL50803_17153 alpha 11-giardin, GL50803_11118 Enolase) based on relative iBAQ values. Directional interactions depicted in black were found in limited cross-linking conditions and confirmed by native co-IP; interactions in dashed gray lines were not confirmed by native co-IP.

Data presented in supplementary table 3 were used to build a UPS and PV/PECs-associated interactome network (Figure 3). Each selected PV/PECs-associated protein is connected to at least one of the other PVs/PECs-associated UPS substrates, with several shared interaction partners (Figure 3 and Supplementary tables 3 and 4). Alpha 1-giardin is the PVs/PECs UPS substrate with the most unique interactors, having tight connections to coiled-coil proteins, NEK kinases, and alpha 6-giardin. Alpha 11-giardin showed reciprocal interactions to all three other selected PV/PECs-localized UPS substrates. Elongation factor 1-alpha and OCT were indiscriminately found across almost all UPS-related datasets in both co-IP conditions and in some wild-type datasets, indicating that abundance rather than specific interaction may account for their presence. For this reason, they are not included in this first interactome and were not selected for further analyses. Furthermore, all proteins found in both wild-type and transgenic samples in cross-linking or native conditions were excluded from further analysis (Supplementary tables 8 and 9, highlighted in gray).

**Figure 4.**
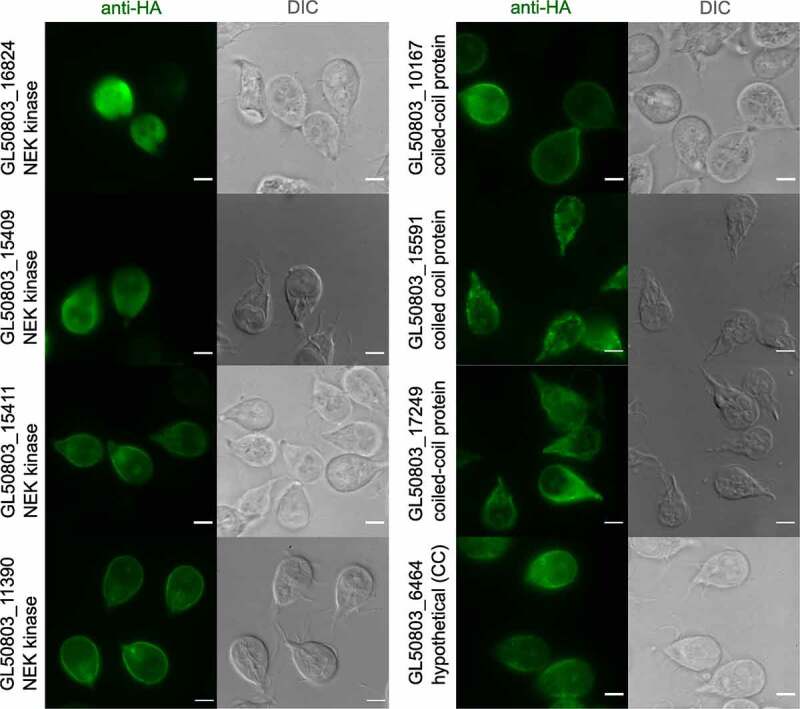
Selected coiled-coil proteins and NEK kinases localize to PV/PECs. Representative widefield light microscopy images of antibody-labeled HA epitope tagged proteins expressed in Giardia trophozoites (anti-HA panels) including DIC images. On the left, GL50803_16824 NEK kinase shows a cytosolic distribution while NEK kinases GL50803_15409, GL50803_15411, and GL50803_11390 show deposition in PV/PECs proximity. On the right the coiled-coil proteins, all showing PV/PECs proximity distribution patterns. GL50803_6464 hypothetical is considered a putative coiled-coil protein (CC) based on *in silico* prediction and shows a PV/PECs distribution pattern as well. Scale bars: 5 µm.

Given their documented roles as scaffolding proteins (NEK kinases) or tethering factors (coiled-coil proteins) [56,57], four NEK kinases (GL50803_15411, GL50803_16824, GL50803_15409, GL50803_11390) all predicted to be catalytically inactive [57], three annotated coiled-coil proteins (ORFs GL50803_10167, GL50803_15591, GL50803_17249) and one uncharacterized protein (GL50803_6464) were selected for further investigation. Homology prediction using the HHpred suite on ORF GL50803_6464 suggests this protein to also be a coiled-coil protein. Antibody labeling and widefield microscopy analysis of transgenic *Giardia* lines expressing epitope-tagged variants of the selected coiled-coil proteins and NEK kinases show reporter deposition at the cell periphery, with the exception of ORF GL50803_16824 NEK kinase, which appears to be predominantly cytosolic (Figure 4).

### An expanded UPS interactome at PV/PECs is dominated by coiled-coil proteins and NEK kinases as accessory proteins

Having determined a molecular complement for UPS substrates at PV/PECs, we sought to expand and validate this protein network by using epitope-tagged variants of the selected coiled-coil proteins and NEK kinases as affinity handles for reverse/reciprocal co-IP experiments in cross-linking conditions. Table 2 lists the 10 most enriched interaction partners for each affinity handle, as measured by relative iBAQ values (%iBAQ) across (at least) two independent biological replicates. Xaa-Pro dipeptidase, alpha 1-giardin, alpha 11-giardin, and enolase are all identified and enriched across datasets derived from co-IP of the eight selected NEK kinases and coiled-coil proteins, thus validating the initial UPS-associated protein interactome. Furthermore, three additional alpha giardins, namely GL50803_7796 alpha 2-giardin, GL50803_14551 alpha-6 giardin, and GL50803_114787 alpha 7.3-giardin, were detected (Table 2).

**Figure 5.**
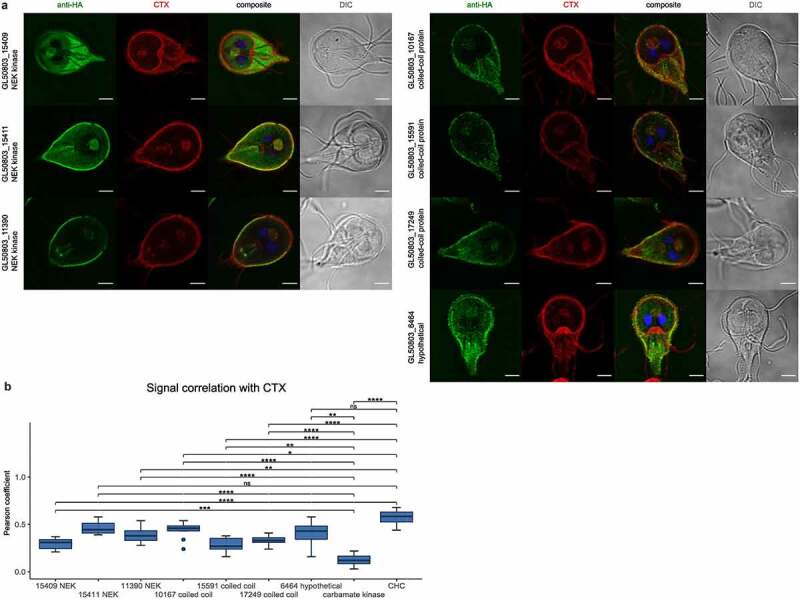
Confocal microscopy and signal overlap analysis confirms selected coiled-coils proteins and NEK kinases at PV/PECs. **(a)** Confocal microscopy images of antibody-labeled HA epitope tagged proteins expressed in Giardia trophozoites (anti-HA panels in green) including co-labelling with the PV membrane marker cholera toxin B, Alexa Fluor™ 594 Conjugate in red (CTX), a composite image of the two panels and nuclei stained with DAPI and a DIC image. **(b)** Signal correlation was quantified calculating Pearson coefficient (*N* = 10). A *t*-test was performed to compare all values to the cytosolic control carbamate kinase and *Gl*chc. Significance levels are indicated by asterisks. The data are displayed as boxplots and n.S. indicates a non-significant difference (*p*-value >0.05). Scale bars: 3 µm.

Widefield microscopy analyses of labeled epitope-tagged variants of the selected coiled-coil proteins and NEK kinases (except ORF GL50803_16824 NEK kinase) show reporter deposition at the cell periphery and bare-zone. Similar to what was previously done for UPS substrates Xaa-Pro dipeptidase, alpha 1-giardin, alpha 11-giardin, and enolase, we refined the subcellular localization analysis for all selected coiled-coil proteins and NEK kinases in co-labeling experiments with CTX, followed by confocal microscopy (Figure 5(a)) and signal overlap analysis using a dedicated macro script (Figure 5(b) and Supplementary data 2). We omitted ORF GL50803_16824 NEK kinase from this analysis due to its predominantly cytosolic deposition pattern.

All seven investigated proteins differ significantly from the cytosolic control carbamate kinase (Figure 2(b), right panel) in their Pearsons coefficient values (Figure 5(b) and Supplementary table S2). GL50803_15411 NEK and GL50803_6464 hypothetical show high signal correlation, not significantly different from correlation values calculated for the *bona fide* PVs/PECs marker *Gl*CHC. Taken together, these and previous data validate the presence of a UPS interactome dominated by selected UPS substrates and associated to a defined set of coiled-coil proteins and NEK kinases at PV/PECs.

## Discussion

### Identification of a UPS-associated protein complex at PV/PECs

*Giardia* is known for its unique subcellular composition with only few endomembrane compartments [16,58–61]. PV/PECs are positioned at the parasite–host interface as the sole gateway into the Giardia cell [18,20,22,62,63] and have been speculated to engage in secretion or excretion [64,65].

In this report, the hypothesis that, although primarily endocytic, PV/PECs may also be involved in release of putative and confirmed virulence factors which are not natively engineered for canonical, i.e. ER-mediated secretion, was formulated. In line with this hypothesis, microscopy analyses show that at least four previously reported putative UPS substrates, namely Xaa-Pro dipeptidase, alpha 1-giardin, alpha 11-giardin, and enolase [28–30], are associated to the PV/PECs organelle system while maintaining a sizable cytosolic pool, as reflected in the signal correlation analysis using CTX as a marker for PV/PECs membranes. Furthermore, validated co-IP data were used to build a PV/PECs-associated UPS interactome which highlights the strong degree of association among the selected PV/PECs-associated UPS substrates themselves as well as interaction with a defined set of coiled-coil proteins and NEK kinases, in both limited-crosslinking and native co-IP experiments. NEK kinases likely play an important role in *Giardia* as they constitute a large fraction of the predicted Giardia kineome [57]. The NEK kinases found in the PV/PECs-associated UPS interactomes are predicted to be catalytically inactive and may have scaffolding functions [57], collaborating with coiled-coil proteins as tethers [66] either within the complex or to maintain the complex at the PV/PECs membrane. Further investigations will be needed to determine the exact role for these protein families at PV/PECs in connection to UPS.

### Could alpha-giardins play a role in PV/PECs membrane traversal?

The co-IP datasets do not report on significant enrichment for transporters and/or components of vesicular carriers, which could have provided clues on how traversal of the PV/PEC membrane and extracellular release of Xaa-Pro dipeptidase, alpha 1-giardin, alpha 11-giardin, and enolase is achieved. On the other hand, a striking feature of the co-IP datasets resides in the apparent enrichment for alpha-giardins as part of the UPS complex at PVs/PECs, including putative UPS substrates alpha 1-giardin and alpha 11-giardin which interact reciprocally and are both indirectly associated to alpha-giardins 2, 6, and 7.3.

Alpha-giardins are Giardia annexin orthologs [67], so far considered cytoskeletal components and shown to carry immunodominant epitopes and to bind glycosaminoglycans [68]. Following calcium-dependent binding of negatively charged (acidic) phospholipids [41], annexins, usually organized in oligomers, can destabilize biological membranes and even insert into them, inducing leakage in liposome-based experiments [44,69,70]. In a recently published review, mammalian annexins are discussed in the context of UPS due to their extracellular release without any detectable canonical secretory signal [44]. Taking these previous reports into account, it is possible to imagine that alpha-giardins may act as both substrates and mediators of PV/PECs-associated UPS in Giardia. This is an intriguing hypothesis which requires further investigation to determine whether this UPS-related mechanism is indeed part of a novel and perhaps unique mode of non-canonical secretion.

## Materials and methods

### Giardia culturing conditions and transfection

*Giardia lamblia* trophozoites of strain WBA C6 (ATCC 50803) were axenically cultured according to previously established protocols [19–21,71]. Cells were grown in standard *Giardia* growth medium at 37°C and passaged every 2–3 days when cultures had reached confluency. Episomal or stable transfection of wild-type parasites with circular or linearized plasmid vectors (pPacV-Integ-based) was performed using electroporation at 350 V, 960 µF, 800 Ω followed by selection with puromycin for 1 week at 50 µg per ml (InvivoGen). Expression of transfected constructs was controlled using immunofluorescence microscopy assays (IFA) and immuno-blotting.

### Construct synthesis

General information on ORFs including transmembrane domain and signal peptide predictions were gathered from the respective gene pages on GiardiaDB (Table 1 and supplementary table 4). ORFs (Supplementary table S1) were amplified via PCR on genomic DNA from *Giardia* lamblia trophozoites of strain WBA C6 (ATCC 50803) and tagged at the C-terminus with a haemagglutinin (HA) – epitope tag using dedicated oligonucleotide pairs (Supplementary table S1). Restriction sites *XbaI* and *PacI* were introduced using the aforementioned oligonucleotides. Resulting amplicons for all constructs were cloned into a modified pPacV-Integ [72] vector (supplementary material). All proteins were expressed under their putative endogenous promoters defined as a fragment 200–250 bps upstream of the ORFs’ start codon. There are two gene copies of Elongation factor 1-alpha present in *Giardia* which differ only by their up- and downstream regions (GL50803_112304 and GL50803_112312) while their coding regions differ only by two nucleotides, which do not affect the predicted protein sequence (Skarin et al. 2011). As these two genes give rise to the same protein, we decided to only further investigate GL50803_112304. The two proteins could not be distinguished by the MS analysis. For this reason, we indicate elongation factor 1-alpha with the number GL50803_112304/-12 in these data sets.

### Immunofluorescence assays and microscopy analysis

Immunofluorescence assays were performed as described in previous studies [19–21,71]. Briefly, transgenic lines were grown to confluency in 12 ml Nunc polystyrene culture tubes (Thermo Fisher Scientific) and then cooled on ice to detach for 30–60 min. Tubes were hit on a soft surface to detach all cells and then centrifuged at 900*g* for 10 min. The cell pellet was washed in PBS (phosphate-buffered saline) and transferred to 1.5-ml Eppendorf tubes where the cells were fixed for a minimum of 1 h or overnight in 3% formaldehyde (Sigma) in PBS. Alternatively, before fixation, cell pellets were resuspended in 40 µl culture medium and incubated with 8 µl of 1 µg/ml cholera toxin B, Alexa Fluor™ 594 Conjugate (Cat. No C22842, Thermo Fisher) at 37°C for 1 h, washed in PBS and then fixed. After fixation, samples were quenched in 0.1 M Glycine in PBS for 5 min before permeabilization in 1 ml of 2% BSA (bovine serum albumin) +0.2% Triton-X-100 in PBS, for 20 min at room temperature. Cells were then incubated with antibodies in 2% BSA+0.1%Triton-X-100 in PBS. Primary antibody: rat-derived monoclonal anti-HA antibody (dilution 1:250; Roche). Secondary antibody: goat-derived anti-Rat IgG (H+L) conjugated to Alexa Fluor 488 (AF488) (dilution 1:250; Thermo Fisher). Samples were incubated at room temperature for 1 h in the dark. After each antibody incubation, samples were washed twice in 1% BSA+0.05% TX-100 in PBS. Cells were then carefully resuspended in *ca*. 30 µl Vectashield (Reactolab) containing 4′-6-diamidino-2-phenylindole (DAPI) as a nuclear DNA label. Cells were imaged at a Leica DM5500 widefield microscope and a Leica SP8 confocal microscope configured with white light lasers, generally at full cell diameter.

Signal correlation analysis using Pearson’s coefficients was performed with Fiji ImageJ for 10 cells per transgenic line on a ROI defined in a standardized fashion by the cell contour (Fiji macro in supplementary data 2; Schindelin et al. 2019). Statistical analysis (*t*-test) was performed in R and visualization in the form of boxplots was done in R and Inkscape vector graphics editor [73]. Pearson values were compared to values from a cytosolic control (carbamate kinase) and a construct previously shown to localize PV/PECs (clathrin heavy chain *Gl*CHC). Significance levels are indicated by asterisks (*p-value ≤0.05, **p-value ≤0.01, ***p-value ≤0.001, ****p-value ≤0.0001). “n.s.” indicates a non-significant difference (*p*-value >0.05).

### Co-immunoprecipitation with limited cross-linking and in native conditions

Protocols for co-immunoprecipitation (co-IP) under limited cross-linking conditions as well as native conditions were adapted from previous studies [19,20]. *Giardia* trophozoites expressing tagged reporter constructs (minimal two independent biological replicates per line) as well as wild-type control WBA cells were grown in one T-25 flask per line, harvested and resuspended in PBS to reach a final volume of 10 ml. For protein cross linking, cells were pelleted and resuspended in 2.25% formaldehyde in PBS and incubated for 30 min at room temperature on a rotating shaker. Cells were then washed in PBS and incubated for 15 min with 10 ml PBS + glycine 100 mM for quenching. Cells were then resuspended in 5 ml RIPA-SDS buffer (50 mM Tris (pH 7.4), 150 mM NaCl, 1% IGEPAL, 0.5% sodium deoxycholate, 0.1% SDS, and 10 mM EDTA. 100 μL) with added 0.1 M phenylmethylsulfonyl fluoride (PMSF) and 50 µl protease inhibitor solution (Sigma). For controlled cell rupture, the resulting cell mixture was sonicated twice for 30 s (60 pulses, 4 output control, 40% duty cycle) in the cold. After sonication, the samples were incubated at 4°C on a rotating shaker for *ca*. 2 h and then centrifuged and filtered (0.2 μm WWPTFE membrane). To the filtrate, 5 ml RIPA-Triton solution (50 mM Tris pH7.4, 150 mM NaCl, 1% IGEPAL, 0.5% Na deoxycholate, 1% Triton x100, 10 mM EDTA) and 40ul anti-HA agarose bead slurry (Thermo Fisher) were added and incubated overnight at 4°C on a rotating shaker. The beads were washed three times in TBS+0.1% Triton X-100 and three times in PBS. Beads were stored dry at −20°C.

For the co-immunoprecipitation in native conditions, four transgenic UPS cargo lines showing a PV/PEC localization (GL50803_11118 Enolase, GL50803_11654 alpha 1-giardin, GL50803_17153 alpha 11-giardin, GL50803_17327 ×aa-Pro dipeptidase), a cytosolic control (GL50803_16453 carbamate kinase) and wild-type control WBA were grown and harvested as described above. The cell pellets were resuspended in 5 ml PBS supplemented with 100 µl PMSF 0.1 M +50 µl protease inhibitor solution before the samples were sonicated, as described above. After sonication 250 µl 20% Triton X-100 was added and the samples were incubated at 4°C on a rotating shaker for *ca*. 2 h. The samples were then centrifuged and filtered (0.2 μm WWPTFE membrane) before the addition of 40 µl anti-HA agarose bead slurry (Thermo Fisher) and incubation overnight. All samples were then subject to mass spectrometry-based protein identification.

### Liquid chromatography mass spectrometry (LC/MS)

Mass spectrometry and protein identification was performed by the Core Facility for Proteomics & Mass Spectrometry of the University of Bern. In a first step, the samples were resuspended in 8 M Urea with 50 mM Tris-HCl at pH 8 and then reduced at 37°C with DTT 0.1 M with 100 mM Tris-HCl at pH 8 and alkylated at 37°C in the dark with IAA 0.5 M and 100 mM Tris-HCl for 30 min. The slurry was then diluted four times with 20 mM Tris-HCl with 2 mM CaCl_2_ before digestion overnight with 100 ng sequencing grade trypsin (Promega). The samples were then centrifuged for peptide extraction from the supernatant which were then subject to liquid chromatography LC-MS (PROXEON coupled to a QExactive mass spectrometer, Thermo Fisher Scientific). µPrecolumn C18 PepMap100 (5 μm, 100 Å, 300 μm × 5 mm, Thermo Fisher Scientific, Reinach, Switzerland) was used to trap the peptides and then they were separated by backflush on a C18 column (5 μm, 100 Å, 75 μm × 15 cm, C18) by applying a 40-min gradient of 5% acetonitrile to 40% in water, 0.1% formic acid, at a flow rate of 350 nl/min. Full Scan was set at a resolution of 70,000, an automatic gain control (AGC) target of 1E06, and a maximum ion injection time of 50 ms. The following settings were applied with the data-dependent method for precursor ion fragmentation: resolution 17,500, AGC of 1E05, maximum ion time of 110 ms, mass isolation window 2 *m*/*z*, collision energy 27, under fill ratio 1%, charge exclusion of unassigned and 1+ ions, and peptide match preferred, respectively. MaxQuant (v. 1.6.14.0) was used for MS data interpretation against a *Giardia lamblia* database (Giardiadb v. 47) using the default MaxQuant settings.

### Co-IP data analysis and availability

MS hits were sorted by their abundance according to the intensity-based absolute quantification (iBAQ) values. Relative abundance was then calculated from the total iBAQ for each protein hit (%iBAQ = iBAQ/ΣiBAQ × 100). Replicates from each tested line were intersected and only hits found in both/all the data sets were further analyzed. The average relative iBAQ was calculated by adding the relative iBAQ values for each hit and dividing them by the number of replicates. Average relative iBAQ was then sorted from highest to lowest. The 10 most enriched hits aside from the bait protein were visualized with Cytoscape (Shannon et al. 2003). Proteomics data are deposited to the ProteomeXchange Consortium via the PRIDE [74] partner repository with the dataset identifiers **PXD035195** (cross-linking co-IP conditions) and **PXD035190** (native co-IP conditions) (supplementary table 5). Data in both P×D035195 and P×D035190 can be freely accessed through http://www.ebi.ac.uk/pride.

## Supplementary Material

Supplemental Material

## Data Availability

All data presented in this manuscript are freely accessible.

## Data deposition

Construct sequence data are deposited online to this location. Proteomics data are deposited to the ProteomeXchange Consortium via the PRIDE (Perez-Riverol et al., 2022) partner repository with the dataset identifiers **PXD035195** (cross-linking co-IP conditions) and **PXD035190** (native co-IP conditions), both freely accessible through http://www.ebi.ac.uk/pride

## References

[1] Balmer EA, Faso C. The road less traveled? Unconventional protein secretion at parasite–host interfaces. Front Cell Dev Biol. 2021;9. DOI:10.3389/fcell.2021.662711 PMC8182054 34109175

[2] Rabouille C, Malhotra V, Nickel W. Diversity in unconventional protein secretion. J Cell Sci. 2012;125:5251–15. 23377655 10.1242/jcs.103630

[3] Rabouille C. Pathways of unconventional protein secretion. Trends Cell Biol. 2017;27:230–240. 27989656 10.1016/j.tcb.2016.11.007

[4] Kim J, Gee HY, Lee MG. Unconventional protein secretion – new insights into the pathogenesis and therapeutic targets of human diseases. J Cell Sci. 2018;131. DOI:10.1242/jcs.213686 29941450

[5] Denny PW, Gokool S, Russell DG, et al. Acylation-dependent protein export in Leishmania*. 2000. Available from: http://www.jbc.org/. 10.1074/jbc.275.15.11017 10753904

[6] Stegmayer C, Kehlenbach A, Tournaviti S, et al. Direct transport across the plasma membrane of mammalian cells of *Leishmania* HASPB as revealed by a CHO export mutant. J Cell Sci. 2005;118:517–527. 15657075 10.1242/jcs.01645

[7] MacLean LM, O’Toole PJ, Stark M, et al. Trafficking and release of Leishmania metacyclic HASPB on macrophage invasion. Cell Microbiol. 2012;14:740–761. 22256896 10.1111/j.1462-5822.2012.01756.x PMC3491706

[8] Möskes C, Burghaus PA, Wernli B, et al. Export of Plasmodium falciparum calcium-dependent protein kinase 1 to the parasitophorous vacuole is dependent on three N-terminal membrane anchor motifs. Mol Microbiol. 2004;54:676–691. 15491359 10.1111/j.1365-2958.2004.04313.x

[9] Nievas YR, Lizarraga A, Salas N, et al. Extracellular vesicles released by anaerobic protozoan parasites: current situation. Cell Microbiol. 2020;22. DOI:10.1111/cmi.13257 32858768

[10] Silverman JM, Chan SK, Robinson DP, et al. Proteomic analysis of the secretome of Leishmania donovani. Genome Biol. 2008;9:R35. 18282296 10.1186/gb-2008-9-2-r35 PMC2374696

[11] Sampaio NG, Emery SJ, Garnham AL, et al. Extracellular vesicles from early stage *Plasmodium falciparum* -infected red blood cells contain PfEMP1 and induce transcriptional changes in human monocytes. Cell Microbiol. 2018;20:e12822. 29349926 10.1111/cmi.12822

[12] Hehl AB, Marti M. Secretory protein trafficking in Giardia intestinalis. Mol Microbiol. 2004;53:19–28. 15225300 10.1111/j.1365-2958.2004.04115.x

[13] Marti M, Hehl AB. Encystation-specific vesicles in Giardia: a primordial Golgi or just another secretory compartment? Trends Parasitol. 2003;19:440–446. 14519581 10.1016/s1471-4922(03)00201-0

[14] Elias EV, Quiroga R, Gottig N, et al. Characterization of SNAREs determines the absence of a typical Golgi apparatus in the ancient eukaryote Giardia lamblia. J Biol Chem. 2008;283(51):35996–36010. DOI:10.1074/jbc.M806545200 18930915 PMC2602913

[15] Faso C, Hehl AB. Membrane trafficking and organelle biogenesis in Giardia lamblia: use it or lose it. Int J Parasitol. 2011;41:471–480. 21296082 10.1016/j.ijpara.2010.12.014

[16] Faso C, Hehl AB. A cytonaut’s guide to protein trafficking in Giardia lamblia. In: Ortega-Pierres MG, editor. Advances in parasitology. Vol. 106. London UK: Academic Press; 2019. pp. 105–127. 31630756 10.1016/bs.apar.2019.08.001

[17] McCaffery JM, Faubert GM, Gillin FD. Giardia lamblia: traffic of a Trophozoite variant surface protein and a major cyst wall epitope during growth, encystation, and antigenic switching. Exp Parasitol. 1994;79:236–249. 7525336 10.1006/expr.1994.1087

[18] Santos R, Ástvaldsson Á, Pipaliya SV, et al. Combined nanometric and phylogenetic analysis of unique endocytic compartments in Giardia lamblia sheds light on the evolution of endocytosis in Fornicata. BMC Biol. 2022;20:*BiorXiv preprint*. 10.1186/s12915-022-01402-3 PMC9490929 36127707

[19] Cernikova L, Faso C, Hehl AB. Phosphoinositide-binding proteins mark, shape and functionally modulate highly-diverged endocytic compartments in the parasitic protist Giardia lamblia. PLOS Pathog. 2020;16:e1008317. 32092130 10.1371/journal.ppat.1008317 PMC7058353

[20] Zumthor JP, Cernikova L, Rout S, et al. Static Clathrin assemblies at the peripheral vacuole—plasma membrane interface of the parasitic protozoan Giardia lamblia. PLOS Pathog. 2016;12:e1005756. 27438602 10.1371/journal.ppat.1005756 PMC4954726

[21] Pipaliya SV, Santos R, Salas-Leiva D, et al. Unexpected organellar locations of ESCRT machinery in Giardia intestinalis and complex evolutionary dynamics spanning the transition to parasitism in the lineage Fornicata. BMC Biol. 2021;19. DOI:10.1186/s12915-021-01077-2. PMC8394649 34446013

[22] Cernikova L, Faso C, Hehl AB. Five facts about Giardia lamblia. PLoS Pathogens. 2018;14:e1007250. 30261050 10.1371/journal.ppat.1007250 PMC6160191

[23] Allain T, Fekete E, Buret AG. Giardia cysteine proteases: the teeth behind the smile. Trends Parasitol. 2019;35:636–648. 31279655 10.1016/j.pt.2019.06.003

[24] Liu J, Ma’ayeh S, Peirasmaki D, et al. Secreted Giardia intestinalis cysteine proteases disrupt intestinal epithelial cell junctional complexes and degrade chemokines. Virulence. 2018;9:879–894. 29726306 10.1080/21505594.2018.1451284 PMC5955458

[25] Ortega-Pierres MG, Argüello-García R. Giardia duodenalis: role of secreted molecules as virulent factors in the cytotoxic effect on epithelial cells. In: Advances in parasitology. Vol. 106. London UK: Academic Press; 2019. pp. 129–169. 31630757 10.1016/bs.apar.2019.07.003

[26] Ortega-Pierres G, Argüello-García R, Laredo-Cisneros MS, et al. Giardipain-1, a protease secreted by Giardia duodenalis trophozoites, causes junctional, barrier and apoptotic damage in epithelial cell monolayers. Int J Parasitol. 2018;48:621–639. 29571981 10.1016/j.ijpara.2018.01.006

[27] Singer SM, Fink MY, Angelova VV. Recent insights into innate and adaptive immune responses to Giardia HHS public access. Adv Parasitol. 2019;106:171–208. 31630758 10.1016/bs.apar.2019.07.004 PMC7086480

[28] Ma’ayeh SY, Liu J, Peirasmaki D, et al. Characterization of the Giardia intestinalis secretome during interaction with human intestinal epithelial cells: the impact on host cells. PLoS Negl Trop Dis. 2017;11:e0006120. 29228011 10.1371/journal.pntd.0006120 PMC5739509

[29] Dubourg A, Xia D, Winpenny JP, et al. Giardia secretome highlights secreted tenascins as a key component of pathogenesis. Gigascience. 2018;7:1–13. 10.1093/gigascience/giy003 PMC5887430 29385462

[30] Davids BJ, Liu CM, Hanson EM, et al. Identification of conserved candidate vaccine antigens in the surface proteome of giardia lamblia. Infect Immun. 2019;87. DOI:10.1128/IAI.00219-19. PMC6529650 30962402

[31] Ahn CS, Kim JG, Shin MH, et al. Comparison of secretome profile of pathogenic and non-pathogenic Entamoeba histolytica. Proteomics. 2018;18:1700341. 10.1002/pmic.201700341 29409117

[32] Tovy A, Tov RS, Gaentzsch R, et al. A new nuclear function of the Entamoeba histolytica glycolytic enzyme enolase: the metabolic regulation of cytosine-5 methyltransferase 2 (Dnmt2) activity. PLOS Pathog. 2010;6:e1000775. 20174608 10.1371/journal.ppat.1000775 PMC2824750

[33] Ghosh AK, Coppens I, Gårdsvoll H, et al. Plasmodium ookinetes coopt mammalian plasminogen to invade the mosquito midgut. Proc Natl Acad Sci U S A. 2011;108:17153–17158. 21949403 10.1073/pnas.1103657108 PMC3193258

[34] Miura N, Kirino A, Endo S, et al. Tracing putative trafficking of the glycolytic enzyme enolase via SNARE-driven unconventional secretion. Eukaryot Cell. 2012;11:1075–1082. 22753847 10.1128/EC.00075-12 PMC3416056

[35] Pancholi V. Multifunctional α-enolase: its role in diseases. Cell Mol Life Sci. 2001;58:902–920. 11497239 10.1007/PL00000910 PMC11337373

[36] Ringqvist E, Palm JED, Skarin H, et al. Release of metabolic enzymes by Giardia in response to interaction with intestinal epithelial cells. Mol Biochem Parasitol. 2008;159:85–91. 18359106 10.1016/j.molbiopara.2008.02.005 PMC3658456

[37] Eckmann L, Laurent F, Langford TD, et al. Nitric oxide production by human intestinal epithelial cells and competition for arginine as potential determinants of host defense against the lumen-dwelling pathogen Giardia lamblia. J Immunol. 2000;164:1478–1487. 10640765 10.4049/jimmunol.164.3.1478

[38] Iharaid S, Miyamoto Y, Le CHY, et al. Conserved metabolic enzymes as vaccine antigens for giardiasis; 2022. DOI: 10.1371/journal.pntd.0010323. PMC9037923 35468132

[39] Stadelmann B, Hanevik K, Andersson MK, et al. The role of arginine and arginine-metabolizing enzymes during Giardia-host cell interactions in vitro; 2013. Available from: http://www.biomedcentral.com/1471-2180/13/256. 10.1186/1471-2180-13-256 PMC4225669 24228819

[40] Adam RD. Giardia duodenalis: biology and Pathogenesis; 2021. DOI:10.1128/CMR. PMC8404698 34378955

[41] Weeratunga SK, Osman A, Hu N-J, et al. Alpha-1 giardin is an annexin with highly unusual calcium-regulated mechanisms. J Mol Biol. 2012;423:169–181. 22796298 10.1016/j.jmb.2012.06.041

[42] Jenikova G, Hruz P, Andersson MK, et al. α1-giardin based live heterologous vaccine protects against Giardia lamblia infection in a murine model. Vaccine. 2011;29:9529–9537. 22001876 10.1016/j.vaccine.2011.09.126 PMC4045459

[43] Weiland MEL, McArthur AG, Morrison HG, et al. Annexin-like alpha giardins: a new cytoskeletal gene family in Giardia lamblia. Int J Parasitol. 2005;35:617–626. 15862575 10.1016/j.ijpara.2004.12.009

[44] Popa SJ, Stewart SE, Moreau K. Unconventional secretion of annexins and galectins. Semin Cell Dev Biol. 2018;83:42–50. 29501720 10.1016/j.semcdb.2018.02.022 PMC6565930

[45] Almagro Armenteros JJ, Tsirigos KD, Sønderby CK, et al. SignalP 5.0 improves signal peptide predictions using deep neural networks. Nat Biotechnol. 2019;37:420–423. 30778233 10.1038/s41587-019-0036-z

[46] Krogh A, Larsson B, von Heijne G, et al. Predicting transmembrane protein topology with a hidden Markov model: application to complete genomes. J Mol Biol. 2001;305:567–580. 11152613 10.1006/jmbi.2000.4315

[47] Franzén O, Jerlström-Hultqvist J, Einarsson E, et al. Transcriptome profiling of Giardia intestinalis using strand-specific RNA-Seq. PLoS Comput Biol. 2013;9:e1003000. 23555231 10.1371/journal.pcbi.1003000 PMC3610916

[48] Tolba MEM, Kobayashi S, Imada M, et al. Giardia lamblia transcriptome analysis using TSS-Seq and RNA-Seq. PLoS ONE. 2013;8:76184. 10.1371/journal.pone.0076184 PMC3792122 24116096

[49] Peirasmaki D, Ma’ayeh SY, Xu F, et al. High Cysteine Membrane Proteins (HCMPs) are up-regulated during Giardia-host cell interactions. Front Genet. 2020;11. DOI:10.3389/fgene.2020.00913. PMC7461913 33014015

[50] Rojas L, Grüttner J, Ma’ayeh S, et al. Dual RNA sequencing reveals key events when different Giardia life cycle stages interact with human intestinal epithelial cells in vitro. Front Cell Infect Microbiol. 2022;12. DOI:10.3389/fcimb.2022.862211 PMC9094438 35573800

[51] Elmendorf HG, Dawson SC, McCaffery JM. The cytoskeleton of Giardia lamblia. Int J Parasitol. 2003;33:3–28. 12547343 10.1016/s0020-7519(02)00228-x

[52] Chavez B, Martinez-Palomo A. Giardia lamblia: freeze-fracture ultrastructure of the ventral disc plasma membrane. J Eukaryot Microbiol. 1995;42:136–141. 7757055 10.1111/j.1550-7408.1995.tb01554.x

[53] Friend DS. THE fine structure of giardia muris. J cell Biol. 1966;29:317–332. 5961344 10.1083/jcb.29.2.317 PMC2106914

[54] Cernikova L, Faso C, Hehl AB. Roles of phosphoinositides and their binding proteins in parasitic protozoa. Trends Parasitol. 2019;35:996–1008. 31615721 10.1016/j.pt.2019.08.008

[55] Corrêa G, Vilela R, Menna-Barreto RFS, et al. Cell death induction in Giardia lamblia: effect of beta-lapachone and starvation. Parasitol Int. 2009;58:424–437. 19703583 10.1016/j.parint.2009.08.006

[56] Gillingham AK, Munro S. Long coiled-coil proteins and membrane traffic. Biochim Biophys Acta Mol Cell Res. 2003;1641:71–85. 10.1016/s0167-4889(03)00088-0 12914949

[57] Manning G, Reiner DS, Lauwaet T, et al. The minimal kinome of Giardia lamblia illuminates early kinase evolution and unique parasite biology. Genome bio. 2011;12. http://genomebiology.com/2011/12/7/R66. 10.1186/gb-2011-12-7-r66 PMC3218828 21787419

[58] Acosta-Virgen K, Chávez-Munguía B, Talamás-Lara D, et al. Giardia lamblia: identification of peroxisomal-like proteins. Exp Parasitol. 2018;191:36–43. 29913139 10.1016/j.exppara.2018.06.006

[59] Tůmová P, Voleman L, Klingl A, et al. Inheritance of the reduced mitochondria of Giardia intestinalis is coupled to the flagellar maturation cycle. BMC Biol. 2021;19. DOI:10.1186/s12915-021-01129-7. PMC8422661 34493257

[60] Soltys BJ, Falah M, Gupta RS. Identification of endoplasmic reticulum in the primitive eukaryote Giardia lamblia using cryoelectron microscopy and antibody to Bip. J Cell Sci. 1996;109:1909–1917. 8832413 10.1242/jcs.109.7.1909

[61] Benchimol M, Souza W. Giardia intestinalis and its endomembrane system*. J Eukaryotic Microbiol. 2022;69. DOI:10.1111/jeu.12893 35148450

[62] Rivero MR, Jausoro I, Bisbal M, et al. Receptor-mediated endocytosis and trafficking between endosomal–lysosomal vacuoles in Giardia lamblia. Parasitol Res. 2013;112:1813–1818. 23315176 10.1007/s00436-012-3253-7 PMC3600075

[63] Abodeely M, DuBois KN, Hehl A, et al. A contiguous compartment functions as endoplasmic reticulum and endosome/lysosome in Giardia lamblia. Eukaryot Cell. 2009;8:1665–1676. 19749174 10.1128/EC.00123-09 PMC2772394

[64] Midlej V, de Souza W, Benchimol M. The peripheral vesicles gather multivesicular bodies with different behavior during the Giardia intestinalis life cycle. J Struct Biol. 2019;207:301–311. 31276754 10.1016/j.jsb.2019.07.002

[65] Moyano S, Musso J, Feliziani C, et al. Exosome biogenesis in the protozoa parasite Giardia lamblia: a model of reduced interorganellar crosstalk. Cells. 2019;8:1600. 31835439 10.3390/cells8121600 PMC6953089

[66] Sztul E, Lupashin V, Sztul E. Role of tethering factors in secretory membrane traffic. Am J Physiol Cell Physiol. 2006;290:11–26. 10.1152/ajpcell.00293.2005 16338975

[67] Morgan RO, Fernández MP. Molecular phylogeny of annexins and identification of a primitive homologue in Giardia lamblia. Mol Biol Evol. 1995;12:967–979. 8524049 10.1093/oxfordjournals.molbev.a040290

[68] Weiland ME-L, Palm JED, Griffiths WJ, et al. Characterisation of alpha-1 giardin: an immunodominant Giardia lamblia annexin with glycosaminoglycan-binding activity. Int J Parasitol. 2003;33:1341–1351. 14527517 10.1016/s0020-7519(03)00201-7

[69] Gerke V, Moss SE. Annexins: from structure to function; 2002. DOI:10.1152/physrev.00030.2001.-Annexins. 11917092

[70] Luecke H, Chang BT, Mailliard WS, et al. Crystal structure of the annexin XII hexamer and implications for bilayer insertion. Nature. 1995;378:512–515. 7477411 10.1038/378512a0

[71] Morf L, Spycher C, Rehrauer H, et al. The transcriptional response to encystation stimuli in Giardia lamblia is restricted to a small set of genes. Eukaryot Cell. 2010;9:1566–1576. 20693303 10.1128/EC.00100-10 PMC2950437

[72] Štefanić S, Morf L, Kulangara C, et al. Neogenesis and maturation of transient Golgi-like cisternae in a simple eukaryote. J Cell Sci. 2009;122:2846–2856. 19622633 10.1242/jcs.049411

[73] R Core Team. R: a language and environment for statistical computing. R Found Stat Comput. 2020;2021:21363.

[74] Perez-Riverol Y, Bai J, Bandla C, et al. The PRIDE database resources in 2022: a hub for mass spectrometry-based proteomics evidences. Nucleic Acids Res. 2022;50:D543–552. 34723319 10.1093/nar/gkab1038 PMC8728295

