# Supplementary material for: A core UPS molecular complement implicates unique endocytic compartments at the parasite–host interface in Giardia lamblia
Source: Virulence. 2023 Feb 13;14(1):2174288. doi: 10.1080/21505594.2023.2174288 (PMC9928461; doi:10.1080/21505594.2023.2174288)
Supplement: Supplemental Material [file KVIR_A_2174288_SM8067.docx]

# Supplementary information

**Supplementary table 1.** List of oligonucleotide pairs used for ORF amplification

[External link](https://docs.google.com/spreadsheets/d/1WWtrFUVpW1N637MuRJlz1ms_B7vP_Aya/edit?usp=share_link&ouid=112012221250134074599&rtpof=true&sd=true)

**Supplementary table 2.** Pearson coefficients calculated in image analysis

[External link](https://docs.google.com/spreadsheets/d/1IPtmDeFKsJK6stlCjYx-OHCLBhL8EyqO/edit?usp=share_link&ouid=112012221250134074599&rtpof=true&sd=true)

**Supplementary table 3.** Top 10 most enriched interactors in co-IP mass spectrometry experiments. Affinity handles for each co-IP dataset are highlighted in orange. Tab 1: results of Co-IP/MS under crosslinking conditions with NEK kinases and coiled-coil proteins highlighted in pale yellow. In the histone reference dataset, other histones are highlighted in pale blue. In the Peroxiredoxin-1 reference dataset for canonical secretion, *bona fide* ER resident proteins are highlighted in pale green. Tab 2: results of co-IP/MS under native conditions with NEK kinases and coiled-coil proteins highlighted in pale yellow. Proteins found in wild-type datasets are highlighted in grey. GN: gene number.

[External link](https://docs.google.com/spreadsheets/d/1p3Ee2dAxdp5wEMHLWOIOBlY3vTbL6ojP/edit?usp=share_link&ouid=112012221250134074599&rtpof=true&sd=true)

**Supplementary table 4.** Annotations for the most enriched proteins found in the interactome network.

[External link](https://docs.google.com/spreadsheets/d/1k1o_M6mKn20jZeMIAIcZbMIJP02mvrqN/edit?usp=share_link&ouid=112012221250134074599&rtpof=true&sd=true)

**Supplementary table 5.** Specifications on samples used for MS analysis of co-IP experiments deposited on PRIDE with identifiers PXD035195 and PXD035190.

[External link](https://docs.google.com/spreadsheets/d/1GSDi8tvwHvORzO-C-nGZW4qrYpGk3kyt/edit?usp=share_link&ouid=112012221250134074599&rtpof=true&sd=true)

**Supplementary table 6.** Original MS datasets derived from coIP experiments performed in cross-linking conditions. Proteins were ordered alphabetically by name/annotation.

[External link](https://docs.google.com/spreadsheets/d/1se2bfr1cjCw1EnO750M4UXJbJj60HXnC/edit?usp=share_link&ouid=112012221250134074599&rtpof=true&sd=true)

**Supplementary table 7.** Original MS datasets derived from co-IP experiments performed in native (non-cross-linking) conditions. Proteins were ordered alphabetically by name/annotation.

[External link](https://docs.google.com/spreadsheets/d/1htbqnsYXAtPEeB-cAjsu2-ONprULjG9z/edit?usp=share_link&ouid=112012221250134074599&rtpof=true&sd=true)

**Supplementary table 8.** Analysis of protein hits from replicates of crosslinking co-IP experiments, dataset intersection and relative %iBAQ calculation, average and st-dev calculation, and ranking.

[External link](https://docs.google.com/spreadsheets/d/1mSho7Hwl98nCwlJry8hPLvtm5xalw4YO/edit?usp=share_link&ouid=112012221250134074599&rtpof=true&sd=true)

**Supplementary table 9.** Analysis of protein hits from replicates of native non-crosslinking co-IP experiments, dataset intersection and relative %iBAQ calculation average and st-dev calculation, and ranking.

[External link](https://docs.google.com/spreadsheets/d/1vxdR7hrEjEcY2vJGJclIrVI6rohenf5q/edit?usp=share_link&ouid=112012221250134074599&rtpof=true&sd=true)

**Supplementary data 1.** Construct sequences in .ape format, freely accessible with the open-source software [APE](https://jorgensen.biology.utah.edu/wayned/ape/) ^93^ and [uploaded to this location](https://drive.google.com/drive/folders/1yJuZHtXW8zKz6vdb8hDTewqakec7GIbY) in both .ape and .txt formats.

**Supplementary data 2**. FIJI Macro for signal correlation analysis on ROI.

[External link](https://drive.google.com/file/d/1hW4NYpvGB08EHAFiN8eM2dXa-O_gxBdW/view?usp=sharing)
